# Analysis of Complications in Postbariatric Abdominoplasty: Our Experience

**DOI:** 10.1155/2015/209173

**Published:** 2015-07-12

**Authors:** Michele Grieco, Eugenio Grignaffini, Francesco Simonacci, Edoardo Raposio

**Affiliations:** Department of Surgical Sciences, Plastic Surgery Division, University of Parma and Cutaneous, Regenerative, Mininvasive and Plastic Surgery Unit, Azienda Ospedaliero-Universitaria di Parma, Via Gramsci 14, 43126 Parma, Italy

## Abstract

Abdominoplasty is one of the most popular body-contouring procedures. It is associated with a significant number of complications: the most common ones are seroma, hematoma, infection, wound-healing problems, and skin flap necrosis. From January 2012 to December 2014, 25 patients (18 women and 7 men) (mean age: 51 years) underwent abdominoplastic surgery at the Plastic Surgery Section, Department of Surgical Sciences, University of Parma, Italy. All patients reported a weight loss between 15 kg and 47 kg. All of the of 25 patients were included in the study; minor and major complications were seen in 17 (68%) and 8 (32%) patients, respectively. The percentage of complications in our patients was as follows: 9 patients with seroma (36%); 4 patients with wound dehiscence with delayed wound healing (16%); 3 cases with hematoma (12%); 2 patients with postoperative bleeding (8%); 1 patient (4%) with an umbilical necrosis; 1 patient (4%) with a deep vein thrombosis; 3 patients with infected seroma (12%); and 2 patients with wound infection (8%). There were no cases of postoperative mortality. The aim of this study is to analyze our complications in postbariatric abdominoplasty.

## 1. Introduction

Abdominoplasty is one of the most popular body-contouring procedures: it is a surgical technique reliable and safe. It is among the top five procedures in aesthetic surgery in the Unites States [1]. However, it is associated with a significant number of complications (32–37.4%) [2, 3]: the most common ones are seroma, hematoma, infection, wound-healing problems, and skin flap necrosis [4, 5]. The aim of postbariatric abdominoplasty was to remove the excess of skin and redundant fat in order to recreate a slim profile [6]. The bariatric population generally presents with a persistently elevated BMI despite having achieved massive weight loss [7]. Obesity is a known risk factor for complications after abdominoplasty [8], but nevertheless an increasing number of abdominoplasties are performed after the boom of bariatric procedures and consequently there are an increasing number of complications [9]. According to Hensel et al., we can define early or minor complications as those occurring in the immediate postoperative period and that were not life-threatening, did not extend hospital stay, and could be managed easily as an outpatient. All the other complications were defined as major ones [2].

## 2. Materials and Methods 

From January 2012 to December 2014, 25 patients (18 women and 7 men) aged between 24 years and 79 years (mean age: 51 years) underwent abdominoplastic surgery at the Plastic Surgery Section, Department of Surgical Sciences, University of Parma, Italy. All patients reported a weight loss between 15 kg and 47 kg (average weight lost: 29 kg), obtained by previous bariatric surgery (14 Pt; 11 F and 3 M) or following a specific diet regimen (11 Pt; 7 F and 4 M). Six months after bariatric surgery, the patients were eligible for functional abdominoplasty. The patients were also advised to stop smoking and female patients were recommended to avoid oral contraception 1 month before surgery. All the procedures were performed with the patient under general anesthesia. The patient is marked in a standing position preoperatively. The skin incision was designed before the intervention as “transverse incision” designed at the superior level of the symphysis pubis and continued laterally to the iliac spines or was designed as inverted “T.” Intraoperatively, the patient was placed in a supine position with the arms abducted 90°. A single-shot second-generation cephalosporin was administered routinely. After the surgical incision of the skin, suprafascial dissection was carried out up to the xiphoid, with a lateral extension depending on the desired magnitude of medial skin shift. At this point, the stage is set to perform myofascial plication, to correct the rectus muscles diastasis. If there were important diastase, incisional hernias, and/or hernias, we prefer to place a polypropylene mesh that had been applied in collaboration with the general surgeons. Following skin resection and reinsertion of the umbilicus, skin closure was performed with absorbable monofilament (Vicryl 2/0 and 3/0 Monosyn). Routinely, two or four suction drains were placed. Postoperatively the drains were removed when fluid collection was <30 mL/24 h, and an abdominal binder was used for 4 weeks postoperatively. For all patients, antibiotic therapy was also administered immediately preoperatively and it was continued for 10 days. Postoperative care included 10 days of treatment with low molecular weight heparin to prevent major complications such as deep vein thrombosis and pulmonary embolism. Social activity was limited for 4 weeks after the discharge.

## 3. Results 

**Figure 1:**
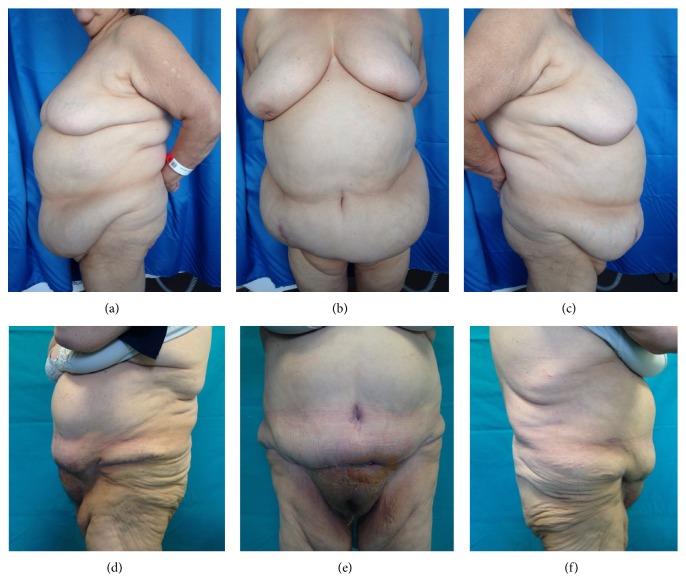
Patient with seroma: (a) + (b) + (c) preoperative case; (d) + (e) + (f) postoperative case.

**Figure 2:**
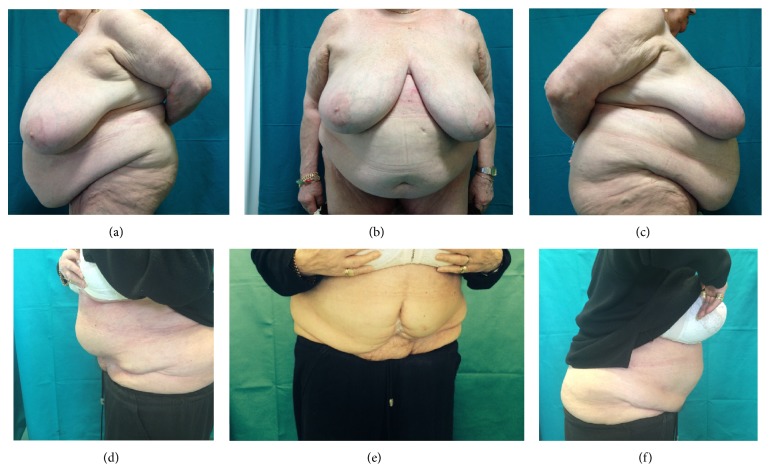
Patient with wound dehiscence: (a) + (b) + (c) preoperative case; (d) + (e) + (f) postoperative case.

**Figure 3:**
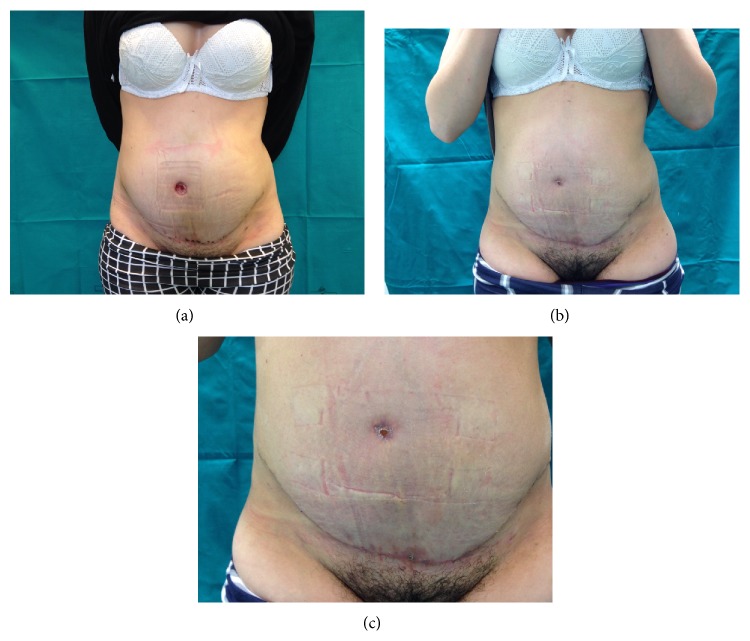
Patient with umbilical necrosis: (a) necrosis of the umbilicus; (b) + (c) navel closed.

Average weight of the 25 patients before surgery was 83,5 kg (range: 58 to 163 kg); average BMI was 31 kg/m^2^ (range 24,77 to 57). All of the 25 patients were included in the study (18 females and 7 males), with a mean age of 51 years at the time of surgery; minor and major complications were seen in 17 (68%) and 8 (32%) patients, respectively. The percentage of complications in our patients was as follows: seroma in 9 patients (36%) (6 F and 3 M) (Figure 1); microbiological swab with antibiogram of the aspirated liquid for the evaluation of any superinfection was performed on all the 9 patients: the replacement of the specific home antibiotic therapy was necessary only for 3 among the 9 patients because of superinfection, in the absence of dehiscence of the wound. In 4 patients, we observed wound dehiscence whit delayed wound healing (16%) (3 F and 1 M) (Figure 2); in all these 4 patients, a punch biopsy from the diastasic wound was done to perform a microbiological analysis of the tissue and the related antibiogram: the home antibiotic therapy was necessarily replaced with a specific postbuffer therapy only in 2 patients (2 F). We observed hematoma in 3 cases (12%) (1 F and 2 M) and postoperative bleeding in 2 patients (8%) (1 F and 1 M) for whom it was necessary to reoperate within 24 hours from the first surgery. Only in 1 female patient have we seen an umbilical necrosis (Figure 3), which was followed by a cycle of dressings until complete healing in a scar well accepted by the patient. Only in 1 male patient have we seen a deep vein thrombosis for which a therapy with low molecular weight heparin for 1 month was necessary (Tables 1-2).

## 4. Discussion

Abdominoplasty or abdominal dermolipectomy is a well-established procedure for improving body contour in aesthetic plastic surgery, with over a 100-year experience since its first publication by Kelly in 1899 [10]. Subsequently, the technique was perfected by Thorek [11] and Pitanguy [12], which, respectively, describe a procedure for preserving the navel and suturing the fascia of the rectus muscles. Considering the increasing popularity of abdominoplasty, there is renewed focus on technical refinements, not only to improve postoperative appearance but also to reduce postoperative complications. A multitude of risk factors has been proposed in the plastic surgery literature, which increased the rate of complications, including smoking [13], obesity [8], hypertension [14], and previous abdominal surgery (gynecologic versus weight-loss procedure) [15]. Obese patients (BMI > 30 kg/m^2^) had an increased total, major, and minor complication rate as compared with nonobese patients (BMI < 30 kg/m^2^) [7, 16]. These obese patients are bariatric patients who remain obese despite prior weight-reduction surgery [7]. Patients' expectations are sometimes underestimated, resulting in dissatisfaction and an increased frequency of follow-up. Our major and minor complication rate were 60% and 11,5%, respectively, which is seemingly consistent with the current literature. Seroma was our most frequent complication [4] according to the literature. It mainly occurs in overweight patients, in those who present a massive weight loss and in patients who present a dead space between the fascia and the abdominal flap after surgery [17], sequential aspirations may be necessary to treat this complication. Seroma is considered a minor complication and therefore is underreported by patients. This complication occurs in 38 to 42% of cases [4, 17, 18]. During abdominoplasty, the use of quilting suture technique described by Baroudi and Ferreira [19] or the use of progressive tension sutures as described by H. Pollock and T. Pollock [20], between the abdominal flap and the rectus abdominis, might help in preventing seroma formation. Similarly, prophylactic drains placement in abdominoplasty has been described as an attempt to reduce seroma formation [21–23]. Hematomas are potential complications of any surgery including abdominoplasty. The frequency of hematoma was less than that of seroma; it occurs in 0.8% to 3% [24] of patients who have undergone abdominoplasty; however, the outcomes were more severe. Hematoma formation was detected by clinical examination, by abdominal ultrasound, and then by aspiration [25]. Abdominal wound-healing problems may occur more readily in previously obese patients [24]. It can arise due to inadequate deep closure or inadvertent straightening-up during the early postoperative period. Different factors can cause the rise of this complication: the presurgery BMI > 25 Kg/m^2^, the concomitant mellitus diabetes, and the tabagism [13, 26]; also the tissue manipulation, associated with surgery, alters the healing process of the wound because of the release of a massive amount of mediators that influence both the coagulation and the complement activity and may therefore compromise the immune system [27, 28]. According to Araco [24, 26] in postbariatric patients, we performed a dissection with diathermocoagulation to reduce the occurrence of postoperative hematomas and wound infections with delayed healing compared with the cold knife. Generally, as long as they are small, conservative wound management and avoiding further stretch on the wound will allow these areas to heal by secondary intention with no significant long-term effect on the overall quality of the scar. This complication is often associated with wound infection [8–13]. In our experience, the most common organisms isolated were* Staphylococcus epidermidis, Staphylococcus aureus, *and* Escherichia coli*. The treatment is appropriate antibiotics, evacuation and drainage of an abscess if present, and debridement and dressing changes. There were no cases of postoperative mortality.

## 5. Conclusion

Postoperative complications were frequent. Patients with complications had a significantly higher reoperation rate, longer hospital stay, and more dissatisfaction. Previous bariatric surgery procedure may play a role similar to so many other widely investigated risk factors such as smoking and BMI, and some categories of patients should require even more attention in the preoperative, intraoperative, and postoperative management. In our experience, often we find patients with great expectations prior to abdominoplasty. For this reason, we believe it is extremely important to inform these patients in the preoperative period about the increased risk of complications that may be incurred with respect to a patient who performs this surgery for aesthetic.

## Figures and Tables

**Table 1:** Minor complications after abdominoplasty in 25 patients.

*Total minor complications *	*17 (68%) *
Hematoma	3 (12%)
Seroma	9 (36%)
Skin necrosis	0
Umbilical necrosis	1 (4%)
Wound dehiscence/delayed wound healing	4 (16%)

**Table 2:** Major complications after abdominoplasty in 25 patients.

*Total major complications *	*8 (32%) *
Abscess	0
Bleeding	2 (8%)
Deep vein thrombosis and pulmonary embolism	1 (4%)
Infected seroma	3 (12%)
Wound infection	2 (8%)
